# Ultrasonic Surgical Aspirator to Treat Deep Infrabony Defects: A New Flapless Minimally Invasive Approach

**DOI:** 10.1155/2018/3612359

**Published:** 2018-07-29

**Authors:** Carlo Ghezzi, Camilla Donghi, Luca Ferrantino, Elena Varoni, Giovanni Lodi

**Affiliations:** ^1^Private Practice, Via Verdi 4, Settimo Milanese, 20019 Milan, Italy; ^2^Department of Oral Rehabilitation, Istituto Stomatologico Italiano, University of Milan, Milan, Italy; ^3^Department of Biomedical, Surgical and Dental Sciences, Università Degli Studi di Milano, Milan, Italy

## Abstract

The primary outcome of the present study was to assess the percentage of pocket closure, and the secondary aim was to evaluate the clinical performance in terms of clinical attachment level (CAL) gain, probing pocket depth (PPD) reduction, and gingival recession (REC) after the use of cavitron ultrasonic surgical aspirator (CUSA) in deep infrabony defects. Fourteen deep infrabony defects in 11 patients who were previously treated with active periodontal therapy followed by one year of supportive periodontal therapy (at least three sessions) were additionally treated by the aid of CUSA. Eighty-six percent of the initial defects (12 out of 14) resulted in a PD < 5 mm, showing complete resolution six months after CUSA treatment, without any adverse event and with negligible pain (VAS from 0 to 3). CUSA showed potential as a method to promote pocket healing, reduce PPD, and increase clinical attachment (*P* < 0.001) in deep infrabony defects. This trial is registered with ClinicalTrials.gov NCT03567161.

## 1. Introduction

Deep periodontal pockets, which are associated with infrabony defects, are specific risk factors for periodontal disease progression and tooth loss [1, 2]. In the past, the interest of researchers on regeneration focused to develop materials as a type of bone substitute and membrane or biological mediator to improve result in tissue regeneration [3–6], but recently, interest has been moving to the tissue management to achieve better result introducing the minimally invasive surgical approaches (MIS) [7–13].

The innovative aspects of the MIS technique are represented by a flap design [12–18] to permit preservation of interdental space, minimizing vertical release in order to obtain adhesion and maturation with slight trauma, together with primary intention wound closure to achieve periodontal tissue regeneration [11, 17–24].

In this context, we have to consider other new studies in which authors define and compare the performance of the minimally invasive nonsurgical technique (MINST) to the minimally invasive surgical approach [25]. MINST has been introduced as a concept that aims at obtaining extensive subgingival debridement with a retention of the preoperative gingival architecture, creating a minimal wound, and gentle handling of the soft and hard tissues to stimulate the formation of a stable blood clot by natural filling of the infrabony defect [25–28].

Cavitron ultrasonic surgical aspirator (CUSA) is a well-known technology that is used in medicine for different purposes; its most frequent applications are in neurosurgery and liver disease [29–31]. CUSA has proven to be effective in biofilm disruption and cell stimulation [32]. The hypothesis is that the employment of CUSA for nonsurgical treatment of infrabony defects, thanks to its abilities to disrupt, fragment, and aspirate granulation tissue, will allow the formation of larger and more stable blood clot.

The purpose of this study was to test CUSA in nonsurgical treatment of infrabony defects to promote pocket closure.

## 2. Materials and Methods

This was a Phase 2 noncontrolled clinical trial performed on patients with infrabony defects to test whether the employment of CUSA for treating periodontal patients.

1. provides benefits in terms of a PD reduction and CAL gain;
2. is comfortable for both the patient and the operator;
3. is free from adverse events.

All subjects included in the study were consecutive periodontal patients attending a private clinic in Settimo Milanese (Milan, Italy) who were treated by two operators (CG and CD) with a similar experience in nonsurgical produce who performed a specific training for CUSA on a periodontal model. They were selected on the basis of the following criteria.

  Inclusion Criteria
  1. Having received a diagnosis of chronic periodontitis (Armitage 1999)
  2. Being treated by full-mouth debridement and supportive periodontal treatment (SPT) in the last year (at least three sessions) by one of the authors
  3. Having at least one residual pocket ≥5 mm with an intrabony component at least ≥2 mm
  Exclusion Criteria
  1. Smoking more than 10 cigarettes per day
  2. Pregnancy
  3. Irregular compliance during SPT in the last year
  4. Systemic conditions or therapies known to affect the healing potential of periodontal tissues (e.g., uncontrolled diabetes, oncological conditions, and immunosuppressant drugs)

All patients were informed on the objective of the studies and provided informed consent.

**Figure 1:**
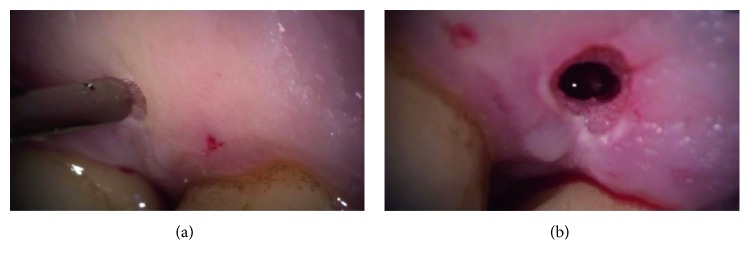
In the case of one or two wall defects, it was possible to use the transgingival approach through the very small access cavity on the basis of the papilla. (a) The sonotrode tip contacting the mucosa surface; (b) the small access after the CUSA treatment.

**Figure 2:**
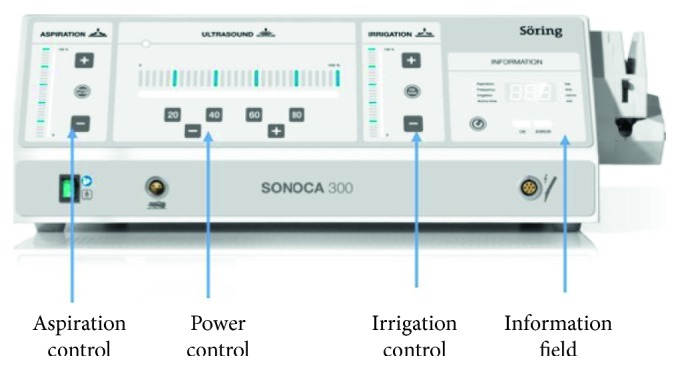
Ultrasound generator: hand-piece recognition and automatic adaptation; three frequencies: 25, 35, and 55 kHz; automatic self-test of all of the important functions predefined power steps or direct adjustment; and optical and acoustical indicator.

**Figure 3 fig3:**
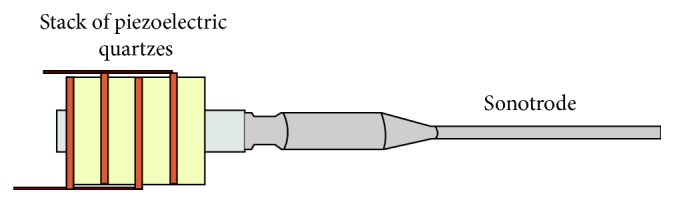
The stack of piezoelectric quartzes transforms the electrical energy of the generator into a longitudinal, mechanical vibration of the sonotrode tip.

**Figure 4:**
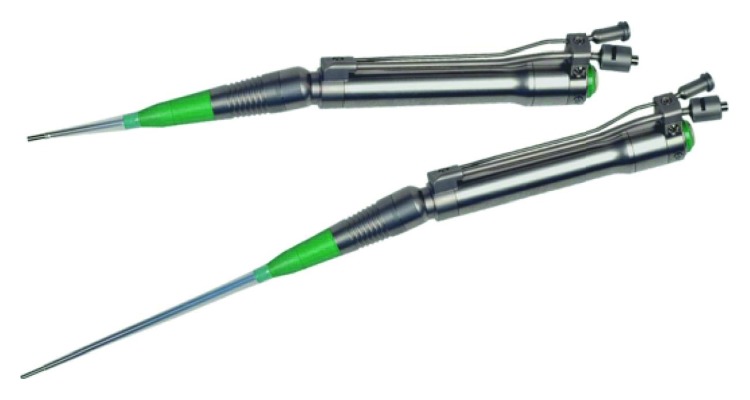
Example of handpieces and sonostrodes.

**Figure 5:**
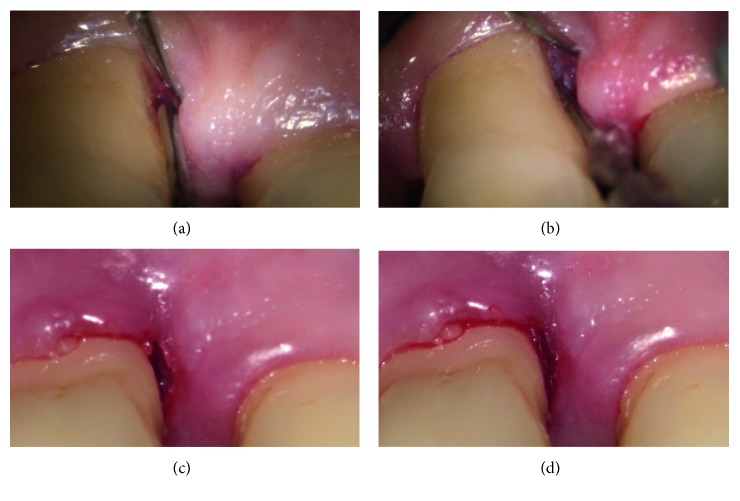
This sequence shows an infrasulcular approach to fragment the tissue. The sonotrode was inserted in a periodontal pocket (a). The tip of the device, placed in contact with the tissues, destroys and emulsifies cells that are irrigated and removed through a built-in suction tube (b). After CUSA treatment, a blood clot fills the target area (c, d).

The clinical procedure was always performed in a single session. Before intervention, all cases received local anaesthesia with 1 : 100,000 mepivacaine. All residual pockets ≥5 mm underwent the following:

1. Ultrasonic debridement: to minimize trauma to the soft tissues, we used piezoelectric devices with specific thin and delicate tips (EMS Electro Medical Systems S.A. Chemin de la Vuarpillière, 31 1260, Lyon, Switzerland).
2. Flapless treatment: according to the anatomy of the osseous sites, the sonotrode (Sonocare 300, Söring GmbH, Justus-von-Liebig-Ring 2-25451 Quickborn, Germany) was inserted both intrasulcularly and transgingivally (smallest tip is 0.8 mm): intrasulcularly, in the cases of three wall defects, and transgingivally in cases of one to two wall defects (Figure 1). The stack of piezoelectric quartzes transforms the electrical energy from the generator into a longitudinal, mechanical vibration of the sonotrode tip (Figures 2 [Fig fig3]–4). When the tip of the sonotrode approaches the tissue, the ultrasonic energy, as a result of the high force of acceleration and cavitation effect, separates cells from the conglomerate of tissues (fragmentation). The fragmented tissue can be aspirated as a semiliquid substance through the sonotrode hole, freeing the defect from the formation of a stable blood clot (Figure 5). The end point is achieving a condition in which the infrabony defect is free from the granulation tissue as if we had performed a surgical technique with the positive biological consequences that were previously described [17–20].

After CUSA treatment, the formation of a stable blood clot was stimulated by avoiding any subgingival rinse.

No medications were prescribed advising the patients to use painkillers (NSAIDs) if they experience postoperative pain.

Subjects were reviewed at 7 days, 15 days, 1 month, 3 months, and 6 months. These sessions included supragingival professional mechanical plaque removal (PMPR) through the use of Erythritol powder plus 14 *μ*m (AIR-FLOW® MASTER-EMS).

Clinical measurements of the defects and X-ray with bite block were taken at baseline and 3 and 6 months. CD and CG acquired all clinical measurements and radiographs, respectively.

The following primary and secondary outcomes were recorded.

  Primary Outcomes
  (i) Pocket closure proportion (PPD < 5 mm)
  (ii) Probing depth (PPD) reduction
  (iii) CAL gain
  (iv) Gingival recession
  Secondary Outcomes
  (i) Comfort and acceptability of the patient during and after the procedure, as measured by interviews, use of painkillers in the following three days, and the visual analogue scale (VAS) after one week;
  (ii) Comfort and convenience of the operator during the procedure, as measured by interviews at the end of the procedure;
  (iii) Adverse events.

Clinical data from all patients were entered into an Excel file and checked for entry errors. Continuous variables were expressed as the mean ± standard deviation (SD). Dichotomous data were expressed as a percentage. The comparison between baseline and 6 months after flapless treatment was performed by applying a Wilcoxon signed-rank test. All calculations were performed using Stata version 11.1 (College Station, TX, USA). The defect was used as a statistical unit, and a *P* value <0.05 was considered statistically significant.

## 3. Results

A total of 14 defects in 11 patients were treated and included in this case series. The demographic and clinical baseline characteristics of the 11 subjects are depicted in Table 1. The average age was 56 ± 8 years. Within the 14 treated defects, five were in the mandible and nine in the maxilla. Seven defects affected the lateral and central incisors, and three were adjacent to premolars and four to molars. The mean PPD at baseline was 8.6 ± 1.5 mm, with an average gingival recession of 2.6 ± 2.1 mm, and therefore, the mean CAL was 11.1 ± 3 mm.  Table 2 shows the characteristics of the 14 infrabony defects included in this study.

Clinical measurements were taken during the last follow-up visit, six months after CUSA treatment (Table 2). The CUSA procedure achieved pocket closure (PPD < 5 mm) in 86% of the defects (12 out of 14).

At that time point, the mean values of PPD, CAL, and REC were 3.9 ± 1.4 mm, 6.8 ± 1.9 mm, and 2.8 ± 1.6 mm, respectively; the differences with baseline data were statistically significant for PPD and CAL (*P* < 0.01). The mean PPD reduction was 4.7 ± 2 mm, and mean CAL gain was 4.3 ± 2.2 mm.

**Figure 6:**
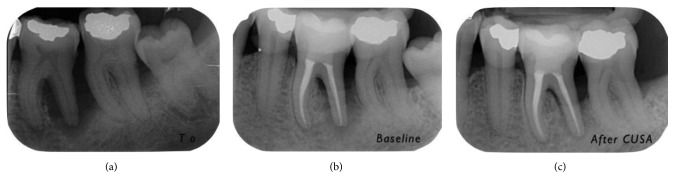
Progression of X-rays showing bone remineralization. Baseline is 12 months after (a) T0. In this case, the PD decreased from 10 mm ((b) baseline) to 4 mm (6 months (c) after CUSA treatment). The CAL gain was 7 mm (from 15 mm to 8 mm).

**Figure 7 fig7:**
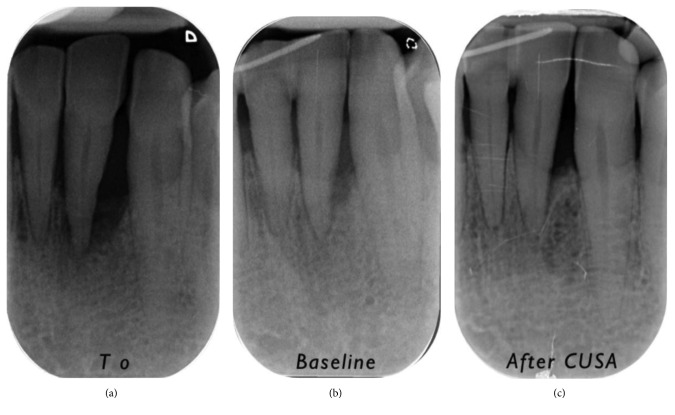
Progression of X-rays showing bone remineralization. Baseline is 12 months after (a) T0. In this case, the PD decreased from 8 mm ((b) baseline) to 3 mm (6 months (c) after CUSA). The CAL gain was 4 mm (from 10 mm to 6 mm).

**Figure 8 fig8:**
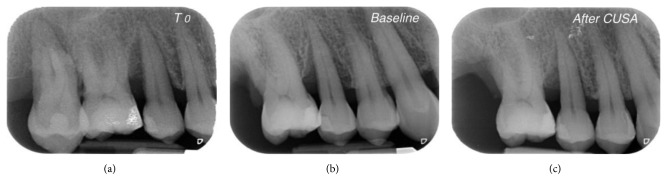
Progression of X-rays showing bone remineralization. Baseline is 12 months after (a) T0. In this case, the PD decreased from 9 mm ((b) baseline) to 4 mm (6 months (c) after CUSA). The CAL gain was 6 mm (from 15 mm to 9 mm).

**Figure 9:**
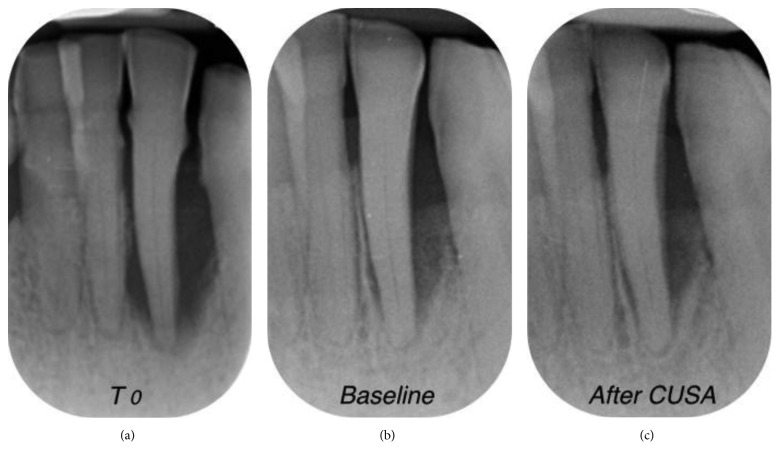
Progression of X-rays showing bone remineralization. Baseline is 12 months after (a) T0. In this case, the PD decreased from 10 mm ((b) baseline) to 4 mm (6 months (c) after CUSA). The CAL gain was 6 mm (from 14 mm to 8 mm).

**Figure 10:**
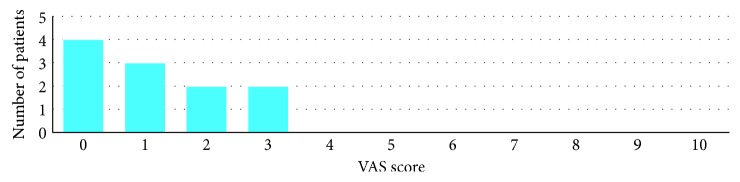
Distribution of the 11 patients by the VAS score after CUSA treatment.

X-rays of the selected defects are presented in Figures 6 [Fig fig7] [Fig fig8]–9. At the end of the procedure, all patients reported negligible discomfort; none took any painkillers in the following week or more. The mean VAS was 1.18 ± 1.11 (the distribution among the study population is shown in Figure 10). The VAS values ranged between 0 and 3; no adverse event was recorded.

## 4. Discussion

Systematic review studies [33, 34] revealed that both conventional nonsurgical and surgical therapies were effective methods for making improvements in terms of CAL gain and PD reduction.

However, in recent studies in which MINST was performed in initially deep pockets (PD > 6 mm), there was a greater CAL gain and PD reduction; the change in these clinical parameters was similar, showing a mean CAL gain of 2.56 mm and PD reduction of 3.13 mm in Ribeiro's study and mean CAL gain of 2.78 and a PD reduction of 3.12 mm in Nibali's study [25, 26]. These data confirm the initial hypothesis of the authors; specifically, the use of minimally invasive strategies in nonsurgical therapy could lead to improvement of results compared to the standard nonsurgical approach [25, 26].

The present study tested a new flapless approach to further improve the results of debridement. For this reason, the starting point of our research was one year after FMUD, followed by repeated sessions of SPT [35].

The primary idea was to eliminate old and capsulated granulation tissue following the chronic process of periodontitis, allowing the defects to have an appropriate and stable new blood clot resulting from a nonsurgical approach. The ability of the surgical aspirator to reach all areas of the defect with Mini tips, fragmenting and aspirating the tissue, makes this instrument particularly suitable to this purpose [32].

Clinical results of the present study showed potential benefits of CUSA as an alternative to current subgingival instruments. We obtained the resolution of 86% of the periodontal pockets (12 out of 14), that is, a reduction of PD at <5 mm, a clinically relevant. CUSA has potential as a method for reducing PD and gaining clinical attachment in deep infrabony defects, showing mean differences of 4.7 ± 1.9 and 4.3 ± 2.1, respectively. This approach may be clearly indicated in patients who are not candidates for traditional surgery.

The tested treatment has been proven to be safe, as no local (i.e., recessions) or systemic adverse event was reported, and no painkillers were necessary for any patient, and pain, as recorded by VAS, was negligible, as demonstrated by the high level of acceptance among patients. In addition, both of the operators who performed the procedures (CG and CD) described it as “simple,” “convenient,” and “rapid,” while indicated, as a major limit of the instrument, the lack of different tips in terms of dimensions and curvatures able to be adapted to the varying tooth anatomy.

The limitations of the present study are the relatively small number of patients enrolled, although this was designed as a preliminary study to test the potential benefits and risks of applying CUSA to gingival and periodontal tissues. Another limitation is that, at present, such instrument is not available for the specific dental application, and thus, we had to design and prepare a small number of ad hoc tips. In addition, the cost of the CUSA is too expensive compared with ultrasonic devices currently used in the routine clinical practice.

## 5. Conclusion

The flapless approach that was used for treating infrabony defects achieved successful outcomes in terms of pocket closure and clinical parameters.

This approach was identified as a promising method to amplify, and in secondary care, the results that are achievable with nonsurgical therapy, promoting less morbidity than any other surgical technique and providing patient satisfaction. This approach requires future randomized control studies to better explain its potential and different application strategies.

## Figures and Tables

**Table 1:** Demographic and clinical baseline variables.

	Patients	
	*n*=11	%
Mean age	56 ± 8 (range 44–67)	

Gender		
Male	8	73
Female	3	27

ASA status		
ASA I	11	100

Periodontal status		
Severe chronic periodontitis	11	100

Race		
Caucasian	11	100

Smoking status		
Nonsmokers	9	82
Light smokers (<10 cigarettes)	2	18

ASA status based on the American Society of Anesthesiologists physical status classification system.

**Table 2:** Clinical variables before and after CUSA treatment.

	Treated defects (*n*=14)		*P* value
	Baseline post-FMUD (mean ± SD)	6-month reevaluation after CUSA treatment (mean ± SD)	
PPD (mm)	8.6 ± 1.5	3.9 ± 1.4	<0.01
CAL (mm)	11.1 ± 3.0	6.8 ± 1.9	<0.01
REC (mm)	2.6 ± 2.1	2.8 ± 1.6	=0.41

FMUD, full-mouth ultrasonic debridement; CAL, clinical attachment loss; REC, recessions; PPD, probing pocket depth. The last column shows the *P* value of the statistical analysis comparing baseline and reevaluation data (statistically significant: *P* < 0.05).

## Data Availability

The data used to support the findings of this study are available from the corresponding author upon request.
